# Supplementary material for: Stability of two-dimensional potential flows using bicomplex numbers
Source: Proc Math Phys Eng Sci. 2022 Jun 8;478(2262):20220165. doi: 10.1098/rspa.2022.0165 (PMC9185835; doi:10.1098/rspa.2022.0165)
Supplement: Supplementary file 1 [file rspa20220165_review_history.pdf]

THE ROYAL SOCIETY
PUBLISHING

PROCEEDINGS A

Stability of two-dimensional potential flows using bicomplex numbers

V. G. Kleine, A. Hanifi and D. S. Henningson

Article citation details
Proc. R. Soc. A **478**: 20220165.
http://dx.doi.org/10.1098/rspa.2022.0165

Review timeline

Original submission:	11 March 2022
Revised submission:	3 May 2022
Final acceptance:	4 May 2022

Note: Reports are unedited and appear as submitted by the referee. The review history appears in chronological order.

Review History

RSPA-2022-0165.R0 (Original submission)

Review form: Referee 1

Is the manuscript an original and important contribution to its field?
Excellent

Is the paper of sufficient general interest?
Excellent

Is the overall quality of the paper suitable?
Excellent

Can the paper be shortened without overall detriment to the main message?
Yes

Do you think some of the material would be more appropriate as an electronic appendix?
No

Do you have any ethical concerns with this paper?
No

Reports © 2022 The Reviewers; Decision Letters © 2022 The Reviewers and Editors; Responses © 2022 The Reviewers, Editors and Authors. Published by the Royal Society under the terms of the Creative Commons Attribution License http://creativecommons.org/licenses/by/4.0/, which permits unrestricted use, provided the original author and source are credited

Recommendation?
Accept as is

Comments to the Author(s)
This is an excellent paper which explores an interesting new connection between the bicomplex number system and the stability analysis of plane potential flows, facilitated by techniques in linear algebra. Rows and streets of point vortices are studied in detail to illustrate the efficacy of the new approach. I found this work to be a very interesting and enjoyable read, and I look forward to future reports on the stability of other vortical systems using this framework.

Review form: Referee 2

Is the manuscript an original and important contribution to its field?
Good

Is the paper of sufficient general interest?
Good

Is the overall quality of the paper suitable?
Good

Can the paper be shortened without overall detriment to the main message?
Yes

Do you think some of the material would be more appropriate as an electronic appendix?
No

Do you have any ethical concerns with this paper?
No

Recommendation?
Accept with minor revision (please list in comments)

Comments to the Author(s)
See Appendix A.

Decision letter (RSPA-2022-0165.R0)

26-Apr-2022

Dear Mr Kleine,

On behalf of the Editor, I am pleased to inform you that your Manuscript RSPA-2022-0165 entitled "Stability of Two-dimensional Potential Flows Using Bicomplex Numbers" has been accepted for publication subject to minor revisions in Proceedings A. Please find the referees' comments below.

The reviewer(s) have recommended publication, but also suggest some minor revisions to your manuscript. Therefore, I invite you to respond to the reviewer(s)' comments and revise your manuscript. Please note that we have a strict upper limit of 28 pages for each paper. Please endeavour to incorporate any revisions while keeping the paper within journal limits. Please note that page charges are made on all papers longer than 20 pages. If you cannot pay these charges you must reduce your paper to 20 pages before submitting your revision. Your paper has been ESTIMATED to be 20 pages. We cannot proceed with typesetting your paper without your agreement to meet page charges in full should the paper exceed 20 pages when typeset. If you have any questions, please do get in touch.

It is a condition of publication that you submit the revised version of your manuscript within 7 days. If you do not think you will be able to meet this date please let me know in advance of the due date.

To revise your manuscript, log into https://mc.manuscriptcentral.com/prsa and enter your Author Centre, where you will find your manuscript title listed under "Manuscripts with Decisions." Under "Actions," click on "Create a Revision." Your manuscript number has been appended to denote a revision.

You will be unable to make your revisions on the originally submitted version of the manuscript. Instead, revise your manuscript and upload a new version through your Author Centre.

When submitting your revised manuscript, you will be able to respond to the comments made by the referee(s) and upload a file "Response to Referees" in Step 1: "View and Respond to Decision Letter". Please provide a point-by-point response to the comments raised by the reviewers and the editor(s). A thorough response to these points will help us to assess your revision quickly. You can also upload a 'tracked changes' version either as part of the 'Response to reviews' or as a 'Main document'.

IMPORTANT: Your original files are available to you when you upload your revised manuscript. Please delete any redundant files before completing the submission process.

When uploading your revised files, please make sure that you include the following as we cannot proceed without these:

1) A text file of the manuscript (doc, txt, rtf or tex), including the references, tables (including captions) and figure captions. Please remove any tracked changes from the text before submission. PDF files are not an accepted format for the "Main Document".

2) A separate electronic file of each figure (tif, eps or print-quality pdf preferred). The format should be produced directly from original creation package, or original software format.

3) Electronic Supplementary Material (ESM): all supplementary materials accompanying an accepted article will be treated as in their final form. Note that the Royal Society will not edit or typeset supplementary material and it will be hosted as provided. Please ensure that the supplementary material includes the paper details where possible (authors, article title, journal name). Supplementary files will be published alongside the paper on the journal website and posted on the online figshare repository (https://figshare.com). The heading and legend provided for each supplementary file during the submission process will be used to create the figshare page, so please ensure these are accurate and informative so that your files can be found in searches. Files on figshare will be made available approximately one week before the accompanying article so that the supplementary material can be attributed a unique DOI.

Alternatively you may upload a zip folder containing all source files for your manuscript as described above with a PDF as your "Main Document". This should be the full paper as it appears when compiled from the individual files supplied in the zip folder.

Article Funder

Please ensure you fill in the Article Funder question on page 2 to ensure the correct data is collected for FundRef (http://www.crossref.org/fundref/).

Media summary

Please ensure you include a short non-technical summary (up to 100 words) of the key findings/importance of your paper. This will be used for to promote your work and marketing purposes (e.g. press releases). The summary should be prepared using the following guidelines:

*Write simple English: this is intended for the general public. Please explain any essential technical terms in a short and simple manner.
*Describe (a) the study (b) its key findings and (c) its implications.
*State why this work is newsworthy, be concise and do not overstate (true 'breakthroughs' are a rarity).
*Ensure that you include valid contact details for the lead author (institutional address, email address, telephone number).

Cover images

We welcome submissions of images for possible use on the cover of Proceedings A. Images should be square in dimension and please ensure that you obtain all relevant copyright permissions before submitting the image to us. If you would like to submit an image for consideration please send your image to proceedingsa@royalsociety.org

Open Access

You are invited to opt for open access, our author pays publishing model. Payment of open access fees will enable your article to be made freely available via the Royal Society website as soon as it is ready for publication. For more information about open access please visit https://royalsociety.org/journals/authors/open-access/. The open access fee for this journal is £1700/$2380/€2040 per article. VAT will be charged where applicable. Please note that if the corresponding author is at an institution that is part of a Read and Publishing deal you are required to select this option. See https://royalsociety.org/journals/librarians/purchasing/read-and-publish/read-publish-agreements/ for further details.

Once again, thank you for submitting your manuscript to Proceedings A and I look forward to receiving your revision. If you have any questions at all, please do not hesitate to get in touch.

Best wishes
Raminder Shergill
proceedingsa@royalsociety.org
Proceedings A

on behalf of
Dr Laurette Tuckerman
Board Member
Proceedings A

Reviewer(s)' Comments to Author:
Referee: 1
Comments to the Author(s)
This is an excellent paper which explores an interesting new connection between the bicomplex number system and the stability analysis of plane potential flows, facilitated by techniques in linear algebra. Rows and streets of point vortices are studied in detail to illustrate the efficacy of the new approach. I found this work to be a very interesting and enjoyable read, and I look forward to future reports on the stability of other vortical systems using this framework.

Referee: 2
Comments to the Author(s)
See Appendix A.

Board Member:
Comments to Author(s):
Your manuscript has now been reviewed by two experts in the field, who both recommend publication. One referee requests minor revisions. PRSA looks forward to receiving a revised version of the manuscript with these revisions.

Decision letter (RSPA-2022-0165.R1)

04-May-2022

Dear Mr Kleine

I am pleased to inform you that your manuscript entitled "Stability of Two-dimensional Potential Flows Using Bicomplex Numbers" has been accepted in its final form for publication in Proceedings A.

Our Production Office will be in contact with you in due course. You can expect to receive a proof of your article soon. Please contact the office to let us know if you are likely to be away from e-mail in the near future. If you do not notify us and comments are not received within 5 days of sending the proof, we may publish the paper as it stands.

As a reminder, you have provided the following 'Data accessibility statement' (if applicable). Please remember to make any data sets live prior to publication, and update any links as needed when you receive a proof to check. It is good practice to also add data sets to your reference list.
Statement (if applicable):

Under the terms of our licence to publish you may post the author generated postprint (ie. your accepted version not the final typeset version) of your manuscript at any time and this can be made freely available. Postprints can be deposited on a personal or institutional website, or a recognised server/repository. Please note however, that the reporting of postprints is subject to a media embargo, and that the status the manuscript should be made clear. Upon publication of the definitive version on the publisher’s site, full details and a link should be added.

You can cite the article in advance of publication using its DOI. The DOI will take the form: 10.1098/rspa.XXXX.YYYY, where XXXX and YYYY are the last 8 digits of your manuscript number (eg. if your manuscript number is RSPA-2017-1234 the DOI would be 10.1098/rspa.2017.1234).

For tips on promoting your accepted paper see our blog post:
https://royalsociety.org/blog/2020/07/promoting-your-latest-paper-and-tracking-your-results/

On behalf of the Editor of Proceedings A, we look forward to your continued contributions to the Journal.

Sincerely,
Raminder Shergill
proceedingsa@royalsociety.org

on behalf of
Dr Laurette Tuckerman
Board Member
Proceedings A

Appendix A

Review of: "Stability of Two-dimensional Potential Flows Using Bicomplex Numbers" by Kleine *et al*

I enjoyed reading this paper, not least because it deals with a certain "subtlety" that, I agree, is not very well appreciated in the scientific community. It is, however, appreciated to certain "cognoscenti" who have wrestled with the same issues themselves, and I include below some additional papers that I encourage the authors to reference and discuss since, in a "review-type" article such as this, I think being as comprehensive as possible in the references/sources is important for its scholarly merit and value to the community. While the paper contains ideas that are not necessarily novel, I think the paper nevertheless serves a useful purpose, and is potentially publishable, with PRSA being a suitable forum for it.

The following articles have adopted a variety of approaches to the very matter dealt with in the present submission:

(1) The following paper is relatively recent one that does *not* avoid the use of complex analysis in a stability calculation of a vortex dynamics problem and should therefore be mentioned:

D.G. Crowdy and M. Cloke "Stability analysis of a class of two-dimensional multipolar vortex equilibria", *Phys. Fluids*, **14**(6), (2002), 1862–1876

Around equation (27) of this paper the authors introduce a device which must be equivalent to the formal use of "bicomplex numbers" in that a distinction is made between complex conjugate quantities in *time* and in *space*.

(2) And in equation (2.15) of

P. J. Baddoo, N. J. Moore, A. U. Oza & D. G. Crowdy, Generalization of waving-plate theory to multiple interacting swimmer, arXiv:2106.09167v1,

a matrix **J** is introduced, again, as a device "to avoid the (potentially significant) confusion of having two distinct imaginary units" (quoting this paper). This device does the same job as an earlier classical paper

T. Y. Wu. Swimming of a waving plate. J. Fluid Mech., 10(03):321–344, 1961

which is careful to introduce two "square roots of minus 1", denoted by "i" (associated with the spatial variable) and "j" (associated with the time variable).

I have not made the comparison carefully myself, but presumably some of the matrices introduced in the present paper do the same job as this matrix **J** of Baddoo *et al.*

(3) That very same device used by Wu is also used in a recent paper

J. C. Hauge & D. G. Crowdy, A new approach to the complex Helmholtz equation with applications to diffusion wave fields, impedance spectroscopy and unsteady Stokes flow, *IMA J. Appl. Math.*, **86**, 1287–1326, (2021)

which is not concerned with linear stability, but with "transform methods" for the complex Helmholtz equation: here too the authors are careful to introduce two "square roots of minus 1", denoted by "i" (associated with the spatial variable) and "j" (associated with the time variable). This paper is worth mentioning as an example of the more general applicability of the authors' ideas on bicomplex numbers to circumstances beyond simple hydrodynamic stability problems.

Finally, it is worth mentioning that about 10 years ago, the famous von Kármán point vortex streets considered here were generalized and a class of *analytical* solutions for von Kármán streets of "hollow vortices" were found:

D.G. Crowdy & C.C. Green, Analytical solutions for von Kármán streets of hollow vortices, *Phys. Fluids*, **23**, 126602, (2011).

I mention this because, to the best of my knowledge, the linear stability of these new analytical solutions has yet to be calculated (a matter of scientific importance in itself) and this would seem to be a tantalizing, and useful, showcase for the bicomplex number formulation outlined here (in contrast, the results of the stability analysis in the present paper are well known, they have just been derived with a novel spin).
